# Radiation-Free Cannulation of the Uterine Artery during Fibroid Embolization Using Fiberoptic Real Shape Technology

**DOI:** 10.1007/s00270-025-04054-2

**Published:** 2025-05-20

**Authors:** Timo A. Auer, Federico Collettini

**Affiliations:** 1 Department of Radiology, Charité-Universitätsmedizin Berlin, Charité Campus Mitte (CCM), Charitéplatz 1, 10117 Berlin, Germany; 2 Clinician Scientist Program, Berlin Institute of Health at Charité-Universitätsmedizin Berlin, Berlin, Germany

**Editor,**

Contemporary radiation safety guidelines underscore the critical necessity of minimizing radiation exposure for both patients and staff [1]. This concern is particularly pronounced among healthcare professionals who routinely perform endovascular procedures, as their cumulative exposure can be substantial. Moreover, the significance of radiation reduction extends to patients, given the substantial rise in the number of endovascular interventions in recent years, driven in part by the rapid expansion of new benign indications including prostates and uterine fibroids, joints, and hemorrhoids enolizations.

Fiberoptic real shape (FORS; Lumiguide, Philips, Amsterdam, Netherlands) technology was recently introduced and received regulatory approval in Europe in December 2019 [2]. FORS utilizes light reflected along optical fibers embedded within wires and catheters to generate real-time, high-fidelity 3D visualization of endovascular devices without the need for fluoroscopy [2]. At present, FORS technology functions as a complementary tool to conventional fluoroscopic techniques rather than a full substitute; nevertheless, it holds considerable potential for substantially reducing radiation exposure during endovascular procedures [2].

**Fig. 1:**
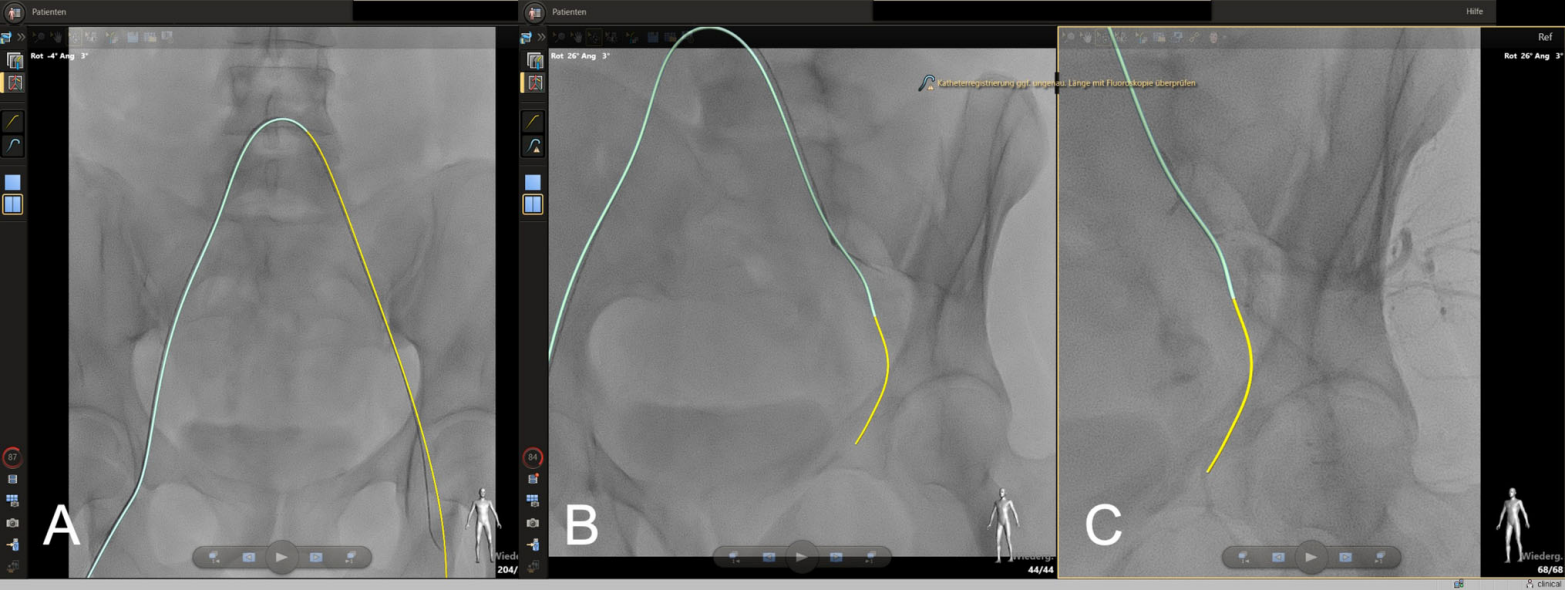
**A** Real-time three-dimensional visualization during the radiation-free cross-over maneuver. The light-emitting wire is visualized as yellow, along the 4F catheter visualized as light blue, which is inserted into the left common femoral artery. **B** Cannulation of the internal iliac artery (p.a.) and **C** in a left anterior oblique reconstruction

**Fig. 2:**
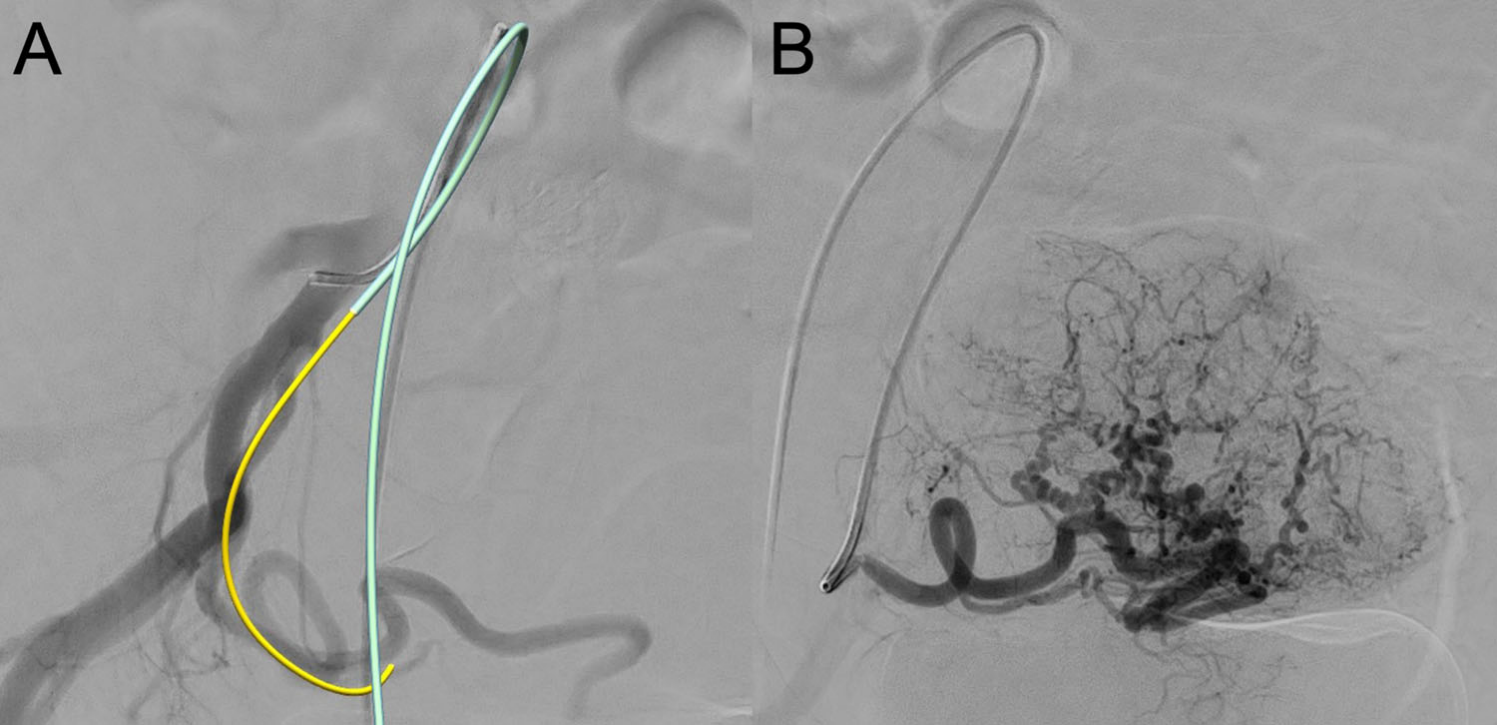
**A** Real-time three-dimensional reconstruction and visualization of a right anterior oblique digital subtraction angiography (DSA) during radiation-free cannulation of the uterine artery. The light-emitting wire is visualized as yellow, along the 4F catheter visualized as light blue, which is inserted into the tortuous right-sided uterine artery. **B** Posterior anterior DSA of the uterine fibroid after radiation-free cannulation of the uterine artery showing a dominant right-sighted supply and no uterine–ovarian anastomoses

Here, we present the case of a 43-year-old female patient with symptomatic uterine fibroids, experiencing heavy menstrual bleeding and pain during menstruation, who was referred for uterine fibroid embolization (UFE). We chose to utilize FORS technology to cannulate the pelvic axis and uterine artery leveraging its advanced real-time 3D visualization capabilities to enhance procedural accuracy while reducing reliance on fluoroscopy. The procedure was performed under local anesthesia and analgesia following a standardized UFE protocol. Vascular access was established using a 4F sheath in the right groin and a 4F macrocatheter in an RIM configuration for vessel probing. After an initial digital subtraction angiography (DSA) overview, the cross-over maneuver was performed, and the left internal iliac artery was cannulated without radiation using FORS technology (Fig. 1). Due to a complex vessel anatomy, a microcatheter was required on the left side, accounting for most of the procedure time. On the contralateral side, the uterine artery was cannulated directly without fluoroscopy. Following DSA from the uterine artery, flow-controlled embolization was carried out using trisacryl gelatin microspheres (500–700 μm, Embosphere®, Merit Medical, South Jordan, Utah, USA) under fluoroscopy until flow stasis in the horizontal part of the uterine artery was achieved. The fluoroscopy time was 10 min and 48 s, with a radiation exposure of 77.4 Gy × cm^2^ in an overweight patient (BMI: 38) (Fig. 2).

Although FORS technology remains relatively unfamiliar in interventional radiology (IR), initial data from vascular surgery on aortic interventions are already available. FORS-based 3D navigation is primarily used for guiding aortic prostheses and their branches. For example, Klaassen et al. (2024) demonstrated a significant reduction in radiation dose during contralateral limb cannulation in endovascular aneurysm repair, while Panuccio et al. (2023) reported shorter catheterization times during complex aortic interventions [3, 4]. Currently, the primary limitation of FORS technology is the available wire length, as it is only offered as a 120 cm, 0.035-inch hydrophilic-coated wire. Nonetheless, IR presents a broad range of potential applications for this technology, potentially driving innovation in the field. As the technology advances, its integration into various IR procedures may enhance workflow efficiency, reduce dependence on traditional imaging techniques, and improve overall procedural safety and effectiveness. At our center, we have begun incorporating FORS into general IR procedures. While we are still navigating the learning curve, the potential to reduce radiation exposure and decrease contrast agent usage appears substantial [5].
